# Poly(N-isopropylacrylamide) Hydrogel for Diving/Surfacing Device

**DOI:** 10.3390/mi12020210

**Published:** 2021-02-19

**Authors:** Jung Gi Choi, Hocheol Gwac, Yongwoo Jang, Christopher Richards, Holly Warren, Geoffrey Spinks, Seon Jeong Kim

**Affiliations:** 1 Center for Self-Powered Actuation, Department of Biomedical Engineering, Hanyang University, Seoul 04736, Korea; oww8989@hanyang.ac.kr (J.G.C.); ghch94@hanyang.ac.kr (H.G.); ywjang@hanyang.ac.kr (Y.J.); 2 ARC Centre of Excellence for Electromaterials Science, Australian Institute for Innovative Materials, University of Wollongong, Wollongong, NSW 2522, Australia; cjr945@uowmail.edu.au (C.R.); hwarren@uow.edu.au (H.W.)

**Keywords:** poly(N-isopropylacrylamide), hydrogel, diving/surfacing device

## Abstract

Underwater robots and vehicles have received great attention due to their potential applications in remote sensing and search and rescue. A challenge for micro aquatic robots is the lack of small motors needed for three-dimensional locomotion in water. Here, we show a simple diving and surfacing device fabricated from thermo-sensitive poly(N-isopropylacrylamide) or a poly(N-isopropylacrylamide)-containing hydrogel. The poly(N-isopropylacrylamide)-containing device exhibited fast and reversible diving/surfacing cycles in response to changing temperature. Modulation of the interaction between poly(N-isopropylacrylamide) chains and water molecules at temperatures above or below the lower critical solution temperature regulates the gel density through the swelling and de-swelling. The gel surfaced in water when heated and sank when cooled. We further showed reversible diving/surfacing cycles of the device when exposed to electrical and ultrasonic stimuli. Finally, a small electrically heated gel was incorporated into a miniature submarine and used to control the diving depth. These results suggest that the poly(N-isopropylacrylamide)-containing device has good potential for underwater remote-controlled micro aquatic robots.

## 1. Introduction

The controlled three-dimensional locomotion of submersible robots, devices and particles is an active area of development with applications including remote sensing [1], cargo transport [2,3], drug release [4], energy harvesting [5,6] and simulation of protocellular behavior [7]. Diving and surfacing are a particular challenge for these systems that have previously been addressed by the generation and trapping of gas for active buoyancy control [2,5,6,8,9,10,11]. In case of gas generation, catalysts or materials for gas production are placed in the device. When they surface, other materials are absorbed into the device and meet the substance already placed in the device. When they meet, they start a chemical reaction and gas is generated as the result of the reaction. Therefore, the inside of the device is filled with generated gas. The gas pushes absorbed water to the outside of the device. Therefore, the device filled with gas loses its density and it surfaces. After the gas-generating reaction is finished, the device absorbs water again and has a higher density which is enough to dive into the water. Another strategy to realize motion of diving and surfacing is heating to expand the volume of the trapped gas. The expanded gas lands the device at the top of the water. After cooling, the volume of gas is decreased and the device dives into the water.

These diving/surfacing processes can be effective for delicate and accurate control by moderating gas generation and expansion, but they are restricted to operation within a specific chemical environment and require the preparation of catalytically active materials with the additional means for containing and trapping a variable gas volume. Diving/surfacing motion depending on the gas requires additional time for a reaction after stimulation and an additional process to repeat the diving/surfacing cycle, such as washing off residue after the reaction, charging the gas-producing materials and trapping gas. Therefore, they need rearrangement after cycles of diving and surfacing. One alternative approach [11] used a smart hydrogel for which the density could be shifted significantly by a small change in temperature. Remote heating of the hydrogel using near infrared (NIR) light irradiation allowed the control of the gel buoyancy in water. The approach is simple and versatile, with operation possible in any aqueous environment, and does not involve any chemical reaction or gaseous species. Here, we further explore the usefulness of density-controlled hydrogels for reversibly diving and surfacing by introducing new modes of heating such as remote ultrasound and direct Joule heating methods. A device fabricated with poly(N-isopropylacrylamide) (PNIPAM) showed a density change resulting from absorbing and releasing water. The absorption and release result from the phase transition of PNIPAM. PNIPAM has two different phases above and below its lower critical solution temperature (LCST, 32 °C), which can be easily reached with heating at room temperature. Therefore, they can be switched by a small change in temperature. Recharge and rearrangement were not required for the device to repeat diving/surfacing cycles, since diving and surfacing occurred without additional materials, and chemical reactions. Thus, the device fabricated with PNIPAM dived and surfaced reversibly and continuously with temperature changes in a relatively low temperature zone.

## 2. Experimental Section

### 2.1. Fabrication of PNIPAM Hydrogel Device

Poly(N-isopropylacrylamide) (PNIPAM), N,N’-methylenebisacrylamide, Irgacure2959 for the photoinitiator and chloroform were purchased from Sigma Aldrich (St. Louis, MO, USA). Amounts of 0.4 g of PNIPAM, 0.04 g of N,N’-methylenebisacrylamide and 0.01 g of photoinitiator were dissolved in 1 mL of chloroform. The solution was mixed with vortexing for 30 min. After mixing, the solution was cast into a rectangular mold and irradiated with UV light (wavelength, λ = 365 nm) for 20 min. The PNIPAM gel was washed after curing to eliminate the residual solution. Washed gel samples were dried at room temperature for more than 12 h.

### 2.2. Fabrication of Electrically Controlled Device

The PNIPAM gel was fabricated by the same process as described in Section 2.1. A nichrome wire with 0.15 mm thickness was used as the heating wire. The pre-gel solution was cast into a rectangular mold that contained a U-shaped nichrome heating wire. UV light (λ = 365 nm) was irradiated for 30 min. Washing and drying were performed with the same process in Section 2.1.

### 2.3. Fabrication of Ultrasonically Controlled Device

The PNIPAM solution was prepared by the same process as describe in Section 2.1. Iron (II, III) oxide (magnetite) which has 50~100 nm of particle size was purchased from Sigma Aldrich (St. Louis, MO, USA). An amount of 0.02 g of magnetite was added to the solution and the solution was mixed by vortexing for 30 min. After that, the solution was irradiated with UV light on a circular mold for 30 min. The washing and drying were carried out with the same process in Section 2.1.

### 2.4. Fabrication of PNIPAM/Carbon Buoyancy Control Device

The N-isopropylacrylamide monomer (NIPAM), N,N’-methylenebisacrylamide for the crosslinker, ammonium persulfate (APS) and tetramethylethylenediamine (TEMED) for the initiator were purchased from Sigma Aldrich, Australia (Macquarie Park, New South Wales, Australia). Carbon foam with 20 pores per inch was purchased from ERG Aerospace (Oakland, CA, USA). At first, 2 g of NIPAM, 0.02 g of crosslinker and 0.2 g of APS were dissolved in 10 mL of deionized water. The solution was cooled in the refrigerator (4 °C) for 30 min. Next, 70 μL of TEMED was added to the solution and it was poured into a 3D printed cylindrical mold. Carbon foam for electrical heating was cut to fit within the mold and the central portion of the foam was removed to form a U shape. Two wires were attached to the carbon foam with a solder. The carbon foam assembly was placed in the mold before pouring the pre-gel solution. Gel polymerization occurred in a refrigerator (4 °C) overnight with a reduced temperature to prevent the phase transition of PNIPAM by heat generated during polymerization. Washing the PNIPAM device was carried out after polymerization to eliminate any residual reactants.

### 2.5. Preparation of Submarine

A remote-controllable submarine was purchased from Create Toys (Shantou, China). The cylindrical PNIPAM/carbon device was inserted into the cavity at the middle part of the submarine. The wires from the PNIPAM/carbon device were connected to the electrical circuit of the submarine which was triggered by the floating button on the remote control. When this button was depressed, a voltage of 4.09 V was applied to the PNIPAM/carbon hydrogel, inducing heating.

### 2.6. Swelling Ratio Measurement

The swelling ratio was calculated by the ratio between the difference of masses of the hydrogel after fully swelling and the dried hydrogel and the mass of the dried hydrogel. Hydrogels stayed in water at room temperature for 1 h for swelling. They were dried at room temperature for 12 h.

### 2.7. Density Measurement

The density of the hydrogel was measured with a pycnometer. PNIPAM gels were fully swelled in each temperature of water for measurement. The water used during measuring was heated to the same temperature as the water for swelling.

### 2.8. Thermal Image

The infrared camera for thermal imaging of the PNIPAM/carbon buoyancy control device was purchased from FLIR (Wilsonville, OR, USA). Calibration was carried out by the built-in program of the camera.

### 2.9. Characterization

Raman spectroscopy was recorded with an NRS-3000 (JASCO, Easton, PA, USA) spectrometer. A laser (λ = 532 nm) was used to measure the Raman spectrum of the dried PNIPAM film. The film for Raman spectroscopy was dried for more than 12 h after washing and inspected for swelling. The contact angle between the PNIPAM film and water was measured by PHOENIX-MT (SEO, Suwon, Korea). The films were swelled in 30 and 40 °C water for one hour to check their surface properties. The water on the surface of each film was eliminated before measuring.

## 3. Results and Discussion

### 3.1. PNIPAM Hydrogel Diving/Surfacing Device

**Figure 1:**
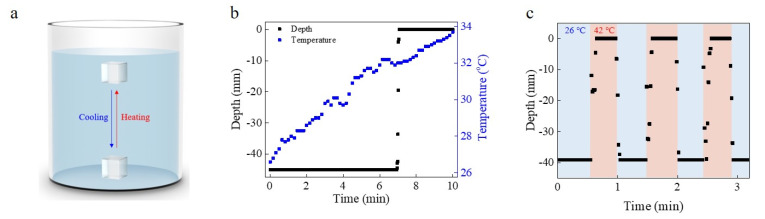
Diving/surfacing actuator fabricated using PNIPAM hydrogel. (**a**) Schematic image of diving/surfacing gel. The gel floats on the water when it is heated above its lower critical solution temperature (LCST) (32 °C) and sinks in the water if the temperature is lower than its LCST. (**b**) Raman spectrum of the device showing that the device consists of PNIPAM. (**c**) Diving/surfacing cycle in water. Diving motion occurred in a cool water bath (26 °C), and surfacing was produced in a hot water bath (42 °C).

Among the promising candidate smart materials, poly(N-isopropylacrylamide) (PNIPAM) and the PNIPAM-based hydrogel can regulate their wettability, water content and volume by the change in their molecular structure that occurs during the phase transition at their lower critical solution temperature (LCST). The popularity of PNIPAM as a stimuli-responsive material is partly due to its convenient LCST that lies near 32 °C in distilled water [12,13,14]. A PNIPAM-based hydrogel-fabricated device for diving and surfacing is illustrated in Figure 1a. The device exploits the change in PNIPAM density that occurs at the LCST so that the material floats in water above its LCST and sinks when the temperature is lower than its LCST. The reversible phase transition that occurs at the LCST means that the diving and surfacing of the device can be reversibly controlled by heating and cooling.

The PNIPAM gel was polymerized in a mold under ultra-violet (UV) light for 20 min Analysis by Raman spectroscopy showed three peaks between 2850 and 3000 cm^−1^, which are indicative of PNIPAM [15] (Figure S1). When the fully dried gel was first placed in water at a temperature below the LCST, it floated. However, after a short period, the gel sank to the bottom of the water bath as it slowly absorbed water and became fully swollen. By contrast, an increase in temperature to above the LCST caused the gel to float. When the water bath temperature was heated from 26.6 to 33.7 °C over 10 min, the device stayed at the bottom until the LCST temperature was reached within the gel and then it started gradually surfacing as the water temperature was increased further (Figure 1b). When the gel temperature exceeded the LCST, the device surfaced a distance of 4.5 cm in 9 s. The phase transition occurring during heating from below to above the LCST sees the release of some water and partial de-swelling of the PNIPAM-based hydrogel.

To test the reversibility of diving and surfacing, the gel was alternatively cycled between hot (42 °C) and cool (26 °C) water baths (Figure 1c and Video S1). Initially, the gel was placed in the cool water bath for 30 s. Thereafter, the device was moved to the hot bath where it started to surface after about 3 s. The gel surfaced at a speed of ~0.55 cm/s in hot water. After reaching the surface, the device maintained floating for 20 s when it was again moved to the cool bath and it started diving right to the bottom. The diving motion occurred over a distance of 3.9 cm to contact the bottom of the bath with an average diving time of 3.67 s of ~1.1 cm/s. The expected terminal speed of the hydrogel for surfacing in water at 42 °C is 1.3 cm/s and for diving at 26 °C, it is 1.0 cm/s each. The expected velocity for surfacing showed a large difference from the observed velocity. One reason for this result is that the PNIPAM hydrogel started surfacing before the density change finished completely. Another reason is that the shape of the hydrogel was not maintained during the diving/surfacing cycle. Therefore, the net force acting on the hydrogel could be different from the calculation, and it resulted in a difference between the expected and measured velocities.

Importantly, the PNIPAM-based hydrogel device showed reversible motion of diving and surfacing with changing the temperature by cooling and heating. Tests on a single gel sample showed unchanged diving and surfacing for 18 complete thermal cycles. The PNIPAM hydrogel could be elongated to almost 32% of its length (Figure S2). Its modulus (2.11 kPa) and strain–stress curve showed the device can be elongated by less strength. Due to the flexibility and the easily deformable properties, the hydrogel can be deformed with less resistance by following the movement of other devices when it is combined with them. Therefore, the device can be used as a density controller in soft robots and aquatic robots with just a small restriction on the actuation for other parts.

### 3.2. Diving/Surfacing Resulted from Swelling

**Figure 2:**
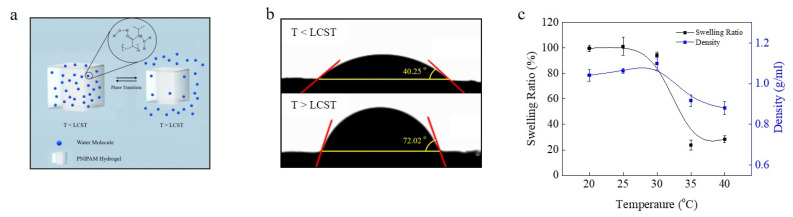
Mechanism of density control during diving/surfacing cycle. (**a**) Schematic image of interaction between PNIPAM molecules and water molecules in each phase of PNIPAM. (**b**) Optical images of contact angle between PNIPAM film and water, which show that PNIPAM becomes less hydrophilic above the phase transition temperature. (**c**) Swelling ratio and density change of PNIPAM device at each temperature.

The change in density by absorbing and releasing water of the PNIPAM-based hydrogel was a result of its phase transition at the LCST. Below the LCST, PNIPAM is hydrophilic with the main interaction with water occurring through hydrogen bonding with the PNIPAM amide nitrogen NH group [16]. Below the LCST, the NH groups in the PNIPAM molecule can be readily accessed by water molecules and interactions between them are not interrupted. However, when the temperature is raised above the LCST, the amide NH groups begin to interact more strongly with the PNIPAM carbonyl oxygens with the exclusion of hydrogen bonding to water [12,13]. The result is that PNIPAM becomes less hydrophilic, leading to loss of water from the gel and a reduction in volume (Figure 2a). To demonstrate the change in the interaction between PNIPAM and water, the contact angle between PNIPAM and water at temperatures above the LCST (42 °C) and below the LCST (26 °C) was measured. As shown in Figure 2b, the contact angle of water on PNIPAM at 26 °C was 40° and 70° at 42 °C, confirming that the hydrogel became less hydrophilic above the LCST.

Figure 2c shows the swelling ratio and the density of the hydrogel at different temperatures. The swelling ratio was near 100% at temperatures below the LCST, whereas it was significantly decreased to almost 20% above the LCST, which resulted in a 40% loss of mass of the gel. In contrast, the density of the water-swollen PNIPAM gel at 30 °C was 1.10 g/mL and it decreased to 0.92 g/mL at 35 °C. This density change resulted because the reduction rate of the swelling ratio of the hydrogel was bigger than that of the volume of the hydrogel (please see the part on the density change in the Supplementary Information). When the temperature changed from 30 to 35 °C, the expected ratio between the decreased density and the former density was 0.79. This was close to the measured value of 0.83. This result shows that the decrease in the swelling ratio was related to the decrease in density. Therefore, the relationship between the change in the swelling ratio and density reflects that the degree of hydration of PNIPAM, which was changed by the phase transition at the LCST, affected the density of the hydrogel.

Water has a density near 1 g/mL, with only a small variation over this temperature range which explains why the device sank when in water below the LCST and floated when heated over the LCST. This sharp change in density made it difficult to control the density of the hydrogel step by step, but the sharp decrease in the density value with a small change at low temperature helped to control the density quickly, easily and safely. Since the PNIPAM hydrogel changed its density by only the swelling ratio, the density could be controlled with just heating and cooling. The value of density showing a clear decrease in a short interval of the temperature resulted in reducing the required time for heating or cooling. The LCST of PNIPAM at low temperature (32 °C) also reduced the danger from largely heating or cooling. Therefore, the PNIPAM hydrogel changed its density in a short time with little temperature change, which is safe for humans.

### 3.3. Electrically and Ultrasonically Controlled Devices

**Figure 3:**
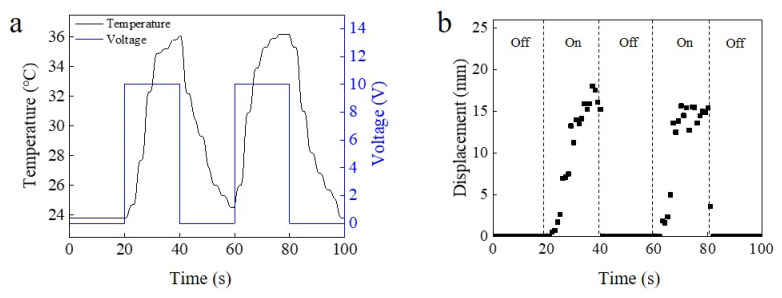
Diving/surfacing cycle of PNIPAM device stimulated by electricity. (**a**) Temperature change of PNIPAM device with inserted heating wire for electrical heating in water. (**b**) Diving/surfacing cycle of the tip of an electrically controlled cantilever device with turning on and turning off of the voltage applier.

The PNIPAM-based hydrogel diving/surfacing device could be heated by various stimuli such as electricity and ultrasound. Figure 3a shows the change in temperature when applying 10 V to a heating wire which was inserted in the gel. This electronically controlled diving/surfacing device was fabricated by inserting a nichrome wire into the PNIPAM hydrogel device. The device was 4 cm long, 1 cm wide and 0.1 cm thick. The nichrome heating wire was inserted as a U shape into the device. The initial temperature of the device in water was 23.8 °C, and it increased to more than 36 °C in 20 s when applying the voltage. Less than 10 s was required for the temperature to reach the LCST (32 °C). The device cooled down to the LCST in less than 5 s when the applied voltage was stopped. The nichrome wire-contained part of the device began to surface when the voltage was turned on (Figure 3b and Video S2) and it moved 1.37 mm in one second.

**Figure 4:**
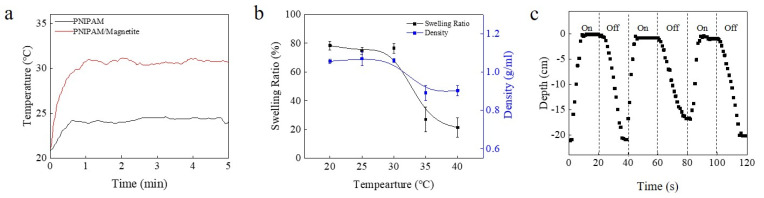
Diving/surfacing device stimulated by ultrasound. (**a**) Temperature change of PNIPAM and PNIPAM/magnetite composite heated by ultrasound. Device can be heated more with magnetite particle than when it consists of just PNIPAM. (**b**) Swelling ratio and density change of PNIPAM/magnetite composite diving/surfacing device. (**c**) Diving/surfacing cycles after the first cycle.

Remote wireless control was also demonstrated using ultrasonic-induced heat. The ultrasonic-controlled device was a circular disc shape with a 24 mm diameter and 1 mm thickness. Magnetite was added to the PNIPAM solution prior to polymerization to facilitate ultrasonic heating [17,18]. According to previous studies, the addition of magnetite particles has no effect on the LCST of PNIPAM [14,19]. When exposed to ultrasonic stimulation, the temperature of the magnetite-containing hydrogel was significantly higher than a similar sized gel made from the neat PNIPAM hydrogel (Figure 4a). The swelling ratio of the magnetite-composited hydrogel device was more than 75% below the LCST and decreased to almost 20% above the phase transition. In addition, the density of the device decreased from 1.05 to 0.90 g/mL during the phase transition (Figure 4b). The diving/surfacing cycle of the ultrasonic-controlled device is shown in Figure S3 and Video S3. Figure 4c indicates the depth of the device located in a glass tube according to ultrasonic stimulation. The ultrasound (44 kHz frequency) was repeatedly generated for 20 s and paused for 20 s. Upon the ultrasonic stimulation, the device rapidly surfaced 2.56 cm and stayed at the surface of the water in a glass tube. The device started to dive as soon as the ultrasound was turned off and stayed at the bottom in the glass tube. Responding to ultrasound, the temperature of the device was increased above the LCST and this state was maintained while the ultrasonic stimulus was kept on. It was observed that the ultrasound-induced device required 21 min to float to the surface during the first application of ultrasound (Figure S3). After this first cycle, however, the diving and surfacing response was rapid, occurring within 20 s because the amount of water absorbed into the device was not as much as that before the first surfacing. This ultrasonic-responsive device presents the possibility of wireless and remote-controllable application, such as for drug delivery.

### 3.4. Remotely Controlled Underwater Vehicle

**Figure 5:**
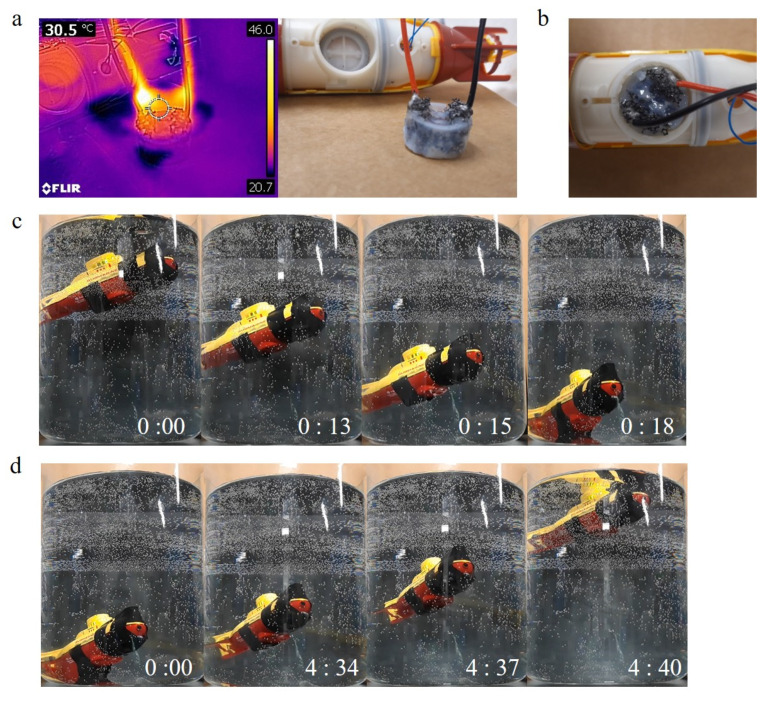
Remotely controllable miniature submarine with adjustable buoyancy using an electrically heated PNIPAM/carbon hydrogel composite. (**a**) Thermal image (left image) of PNIPAM/carbon device when supplied with 4.09 V for five minutes. The right image shows the PNIPAM/carbon gel with electrical connections and before insertion in the submarine. (**b**) PNIPAM/carbon buoyancy control device placed in the cavity at the underside of the submarine. Note that the red protective shell of the submarine has been removed to show the hydrogel composite and connecting wires. (**c**) Optical images of the submarine in water during sinking. (**d**) Images of submarine during floating. A pre-heating period of 4 min and 34 s was required to initiate floating which was then completed within 6 s.

Figure 5 shows an example application where the diving depth of a miniature submarine could be controlled using an electrically heated PNIPAM gel. The radio-controlled submarine was modified by removing its piston-driven bellows system used for diving and surfacing. The cylindrical cavity remaining after removal of the bellows was filled with an electrically conductive PNIPAM gel (Figure 5b) and the connecting wires used for driving the piston were re-directed to enable electrical heating of the gel (Figure 5a). Finally, the buoyancy of the submarine was adjusted by the addition of weights so that the submarine was negatively buoyant in a water bath at 22 °C.

To ensure rapid and even Joule heating, the gel was prepared as a composite of the PNIPAM hydrogel and conductive carbon foam. The submarine’s on-board battery supplied 4.09 V when the remote controller’s surfacing button was depressed. Heat was generated via electrical conduction through the carbon foam and the temperature of the composite was raised to above the LCST of PNIPAM. The thermal image in Figure 5a shows the state of the device after 5 min of electrical heating by the voltage supplied from the on-board battery. The maximum temperature observed near the electrical wires was 42 °C, whereas the temperature at the middle of the PNIPAM/carbon device was 31 °C, inferring that the temperature of the PNIPAM gel was raised close to its LCST.

The electronically controlled diving and surfacing of the submarine are shown in Figure 5c,d. With the addition of 16.23 g of added mass, the submarine sank to the bottom of the water tank at 22 °C (Figure 5c). When the remote control was activated, the battery of the submarine applied the voltage to the PNIPAM/carbon composite gel, and the device started to be heated. The voltage was applied continuously while the button was pushed. After 4 min and 34 s, the submarine started floating. It needed 16 s to finish floating and to reach the surface of the water. The submarine remained floating after the remote control switch was deactivated, allowing the composite gel to cool. The submarine sank down again after around 30 min of swelling. The demonstration illustrates how the density of the PNIPAM hydrogel can be used to alter the buoyancy of a larger structure from negative to positive using electrical heating. However, the response time of this demonstration device is impractically slow, and a re-design of the gel dimensions is necessary to ensure shorter response times for heat and mass transport. The structural approach can be an effective strategy to shorten the response time because the changes in the density of the PNIPAM hydrogel result from absorbing and releasing water. Thus, the amount of water which moves to inside or outside of the device largely affects the response time. A large surface area is advantageous to absorb and release a large amount of water rapidly. Therefore, a shorter response time can be realized by a structure with a large surface area.

## 4. Conclusions

In conclusion, PNIPAM hydrogel devices for cyclic diving/surfacing showed reversible sinking and floating in response to temperature changes. The phase transition of PNIPAM induced the exchange of the water between the hydrogel and the surrounding bath, which resulted in the change in density. The device needed no additional processes such as producing gas, washing off residue and replenishment of fuel. Further, the phase transition of PNIPAM occurred at relatively low temperature. Therefore, the danger from heating the device is reduced and this helps the device secure safety in being applied in soft robotics. Moreover, electronically controlled heating suggests the application of wireless underwater vehicles and swimming robots, as demonstrated with a remote-controlled miniature submarine. The simple mechanism for buoyancy control may be further downsized for use in micro aquatic robots.

## Supplementary Materials

The following are available online at https://www.mdpi.com/2072-666X/12/2/210/s1, Figure S1: Raman Spectrum of PNIPAM hydrogel, Figure S2: The first cycle of PNIPAM/magnetite composite, Video S1: Diving/Surfacing cycle of PNIPAM hydrogel, Video S2: The cycle of electrically controlled device, Video S3: The cycle of PNIPAM/magnetite composite, Video S4: The cycle of remote controlled submarine.
