# Supplementary material for: Poly(N-isopropylacrylamide) Hydrogel for Diving/Surfacing Device
Source: Micromachines (Basel). 2021 Feb 19;12(2):210. doi: 10.3390/mi12020210 (PMC7921990; doi:10.3390/mi12020210)
Supplement: Supplementary file 1 [file micromachines-12-00210-s001.zip › micromachines-1107478 Supplementary.docx]

**Poly(n-isopropylacrylamide) Hydrogel for Diving/Surfacing Device**

**Jung Gi Choi^1^, Hocheol Gwac^1^, Yongwoo Jang^1^, Christopher Richards^2^, Holly Warren^2^, Geoffrey. M. Spinks^2^*, Seon Jeong Kim^1^***

^1^ Center for Self-powered Actuation and Department of Biomedical Engineering, Hanyang University, Seoul 04736, South Korea..

^2^ Australian Institute for Innovative Materials, ARC Centre of Excellence for Electromaterials Science, University of Wollongong, Wollongong, New South Wales 2522, Australia.

E-mail: sjk@hanyang.ac.kr; gspinks@uow.edu.au


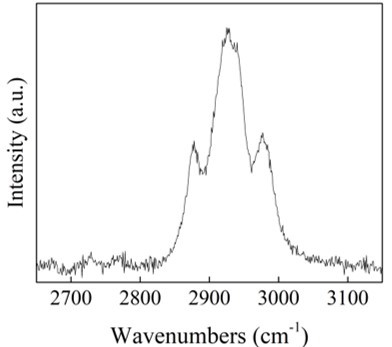

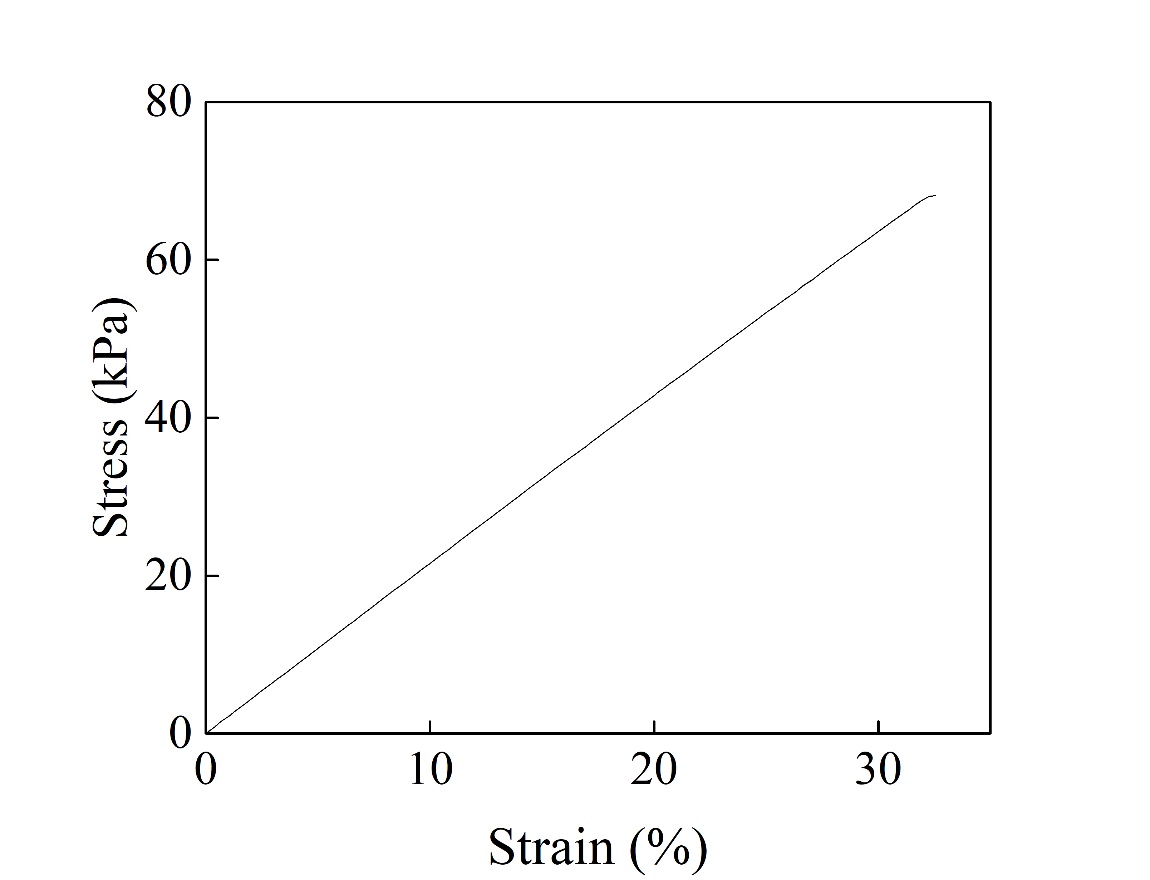


**Figure S1**. Raman spectrum of PNIPAM hydrogel.

**Figure S2**. The strain-stress curve of PNIPAM hydrogel. The modulus is 2.11 kPa and it is broken at almost 32 % strain.

**
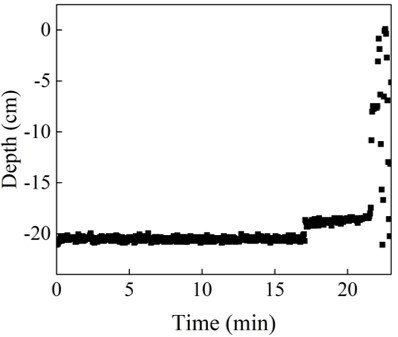
**

**Figure S3**. The first cycle of diving/surfacing of PNIPAM/Magnetite composite stimulated by ultrasound

**Velocity of Hydrogel**

Net force effecting the hydrogel in water is summation of gravity, buoyancy, and drag force. When all forces are balanced, net force is 0 N. Therefore, it can be written as formula (a_1_) and (a_2_).

When the hydrogel diving,

B + F_d_ - mg = 0 (a_1_)

When the hydrogel surfacing,

B - F_d_ - mg = 0 (a_2_)

B is the buoyancy force, F_d_ is drag force, m is the mass of hydrogel, and g is gravitational acceleration.

When D_f_ is the density of fluid, D_p_ is the density of hydrogel, V is the volume of hydrogel, v is velocity of hydrogel, A is cross sectional area of the hydrogel, and C_d_ is drag coefficient,

Buoyancy can be calculated by B = D_f_Vg, and drag force also be calculated as D_f_v^2^AC_d_/2.

Therefore, when diving of the hydrogel,

D_f_Vg + D_f_v^2^AC_d_/2 – mg = 0

v^2^ = 2(mg- D_f_Vg)/D_f_AC_d_

= 2g(m- D_f_V)/D_f_AC_d_

v = {2g(m- D_f_V)/D_f_AC_d_}^1/2^

Because m = D_p_V,

v = {2g(D_p_V- D_f_V)/ρ_f_AC_d_}^1/2^

= {2gV(D_p_ - D_f_)/D_f_AC_d_}^1/2^

When surfacing of hydrogel

D_f_Vg - D_f_v^2^AC_d_/2 – mg = 0

v^2^ = 2(D_f_Vg-mg)/D_f_AC_d_

= 2g(D_f_V-m)/D_f_AC_d_

v = {2g(D_f_V-m)/D_f_AC_d_}^1/2^

Because m = D_p_V,

v = {2g(D_f_V- D_p_V)/D_f_AC_d_}^1/2^

= {2gV(D_f_ – D_p_)/D_f_AC_d_}^1/2^

**Swelling Ratio**

Swelling ratio of hydrogels were calculated by formula (b).

SR = (m_s_ -m_d_)/m_d_ (b)

SR is the swelling ratio, m_s_ is the mass of the hydrogel after fully swelling, m_d_ is the mass of dried hydrogel.

**Density of PNIPAM Hydrogel**

Density of PNIPAM gel was measured by pycnometer. The density was measured by formula (c).

D_p_ = D_w_m_p_/(m_1_-m_2_+m_p_) (c)

The D_p_ is density of PNIPAM hydrogel, D_w_ is density of water, m_p_ is the mass of fully swelled PNIPAM gel. The m_1_ is the mass of pycnometer filled with water, and m_2_ is the mass of the pycnometer filled with gel and water.

Formula (c) is induced as followed:

When V_p_ is the volume of PNIPAM gel,

1. D_p_ = m_p_/V_p_

When PNIPAM gel placed in full filled pycnometer, the same volume of water is pushed out. Therefore, when V_w_ is the volume of pushed water, m_w_ is the mass of pushed water, and m_0_ is the mass of pycnometer without water and gel, V_p_ is calculated as followed:

1. V_p_ = V_w_

= m_w_/D_w_

={(m_1_-m_0_)-(m_2_-m_0_-m_p_)}/D_w_

= (m_1_-m_2_+m_p_)/D_w_

1. D_p_ = m_p_/V_p_

= m_p_/{(m_1_-m_2_+m_p)_/D_w_}

=D_w_m_p_/(m_1_-m_2_+m_p_)

**Density Change of Hydrogel**

Density of hydrogel was controlled by swelling ratio change. Relationship between swelling ratio change and density change is induced as followed:

when, SR is the swelling ratio, D is the density of swollen hydrogel, V is the volume of swollen hydrogel, m_d_ is the mass of dried hydrogel, m_s_ is the mass of hydrogel after swelling,

1. SR= (m_s_-m_d_)/m_d_

= m_s_/m_d_ – 1

Therefore, m_s_ = m_d_(SR+1)

1. D = m_s_/V

= m_d_(SR+1)/V

1. D_2_/D_1_ = {m_d_(SR_2_+1)/V_2_}/{m_d_(SR_1_+1)/V_1_}

= (SR_2_+1)V_1_/(SR_1_+1)V_2_

= {(SR_2_+1)/(SR_1_+1)} * (V_1_/V_2_)

When D_2_/D_1_ < 1, the density is decreased.

Therefore, if (SR_1_+1)/(SR_2_+1) > V_1_/V_2_, the density is decreased after swelling ratio change.

**Cost of the device**

0.4 g of PNIPAM(21.036 USD), 0.04 g of N,N’-methylenebisacrylamide(0.088 USD), 0.01 g of irgacure 2959(0.046 USD), and 1 ml of chloroform(0.131 USD) were used to fabricate 80 mm^3^ of the device. Therefore, the cost of 100 mm^3^ of the device is 26.62 USD. Almost 720 mm^3^ of PNIPAM gel was used at experiment in Figure 5.
